# Negatively-Marked MCQ Assessments That Reward Partial Knowledge Do Not Introduce Gender Bias Yet Increase Student Performance and Satisfaction and Reduce Anxiety

**DOI:** 10.1371/journal.pone.0055956

**Published:** 2013-02-20

**Authors:** A. Elizabeth Bond, Owen Bodger, David O. F. Skibinski, D. Hugh Jones, Colin J. Restall, Edward Dudley, Geertje van Keulen

**Affiliations:** 1 Institute of Life Science, College of Medicine, Swansea University, Swansea, Wales, United Kingdom; 2 Advanced Professional Training in Bioscience, College of Science, Swansea University, Swansea, Wales, United Kingdom; University of Westminster, United Kingdom

## Abstract

Multiple-choice question (MCQ) examinations are increasingly used as the assessment method of theoretical knowledge in large class-size modules in many life science degrees. MCQ-tests can be used to objectively measure factual knowledge, ability and high-level learning outcomes, but may also introduce gender bias in performance dependent on topic, instruction, scoring and difficulty. The ‘Single Answer’ (SA) test is often used in which students choose one correct answer, in which they are unable to demonstrate partial knowledge. Negatively marking eliminates the chance element of guessing but may be considered unfair. Elimination testing (ET) is an alternative form of MCQ, which discriminates between all levels of knowledge, while rewarding demonstration of partial knowledge. Comparisons of performance and gender bias in negatively marked SA and ET tests have not yet been performed in the life sciences. Our results show that life science students were significantly advantaged by answering the MCQ test in elimination format compared to single answer format under negative marking conditions by rewarding partial knowledge of topics. Importantly, we found no significant difference in performance between genders in either cohort for either MCQ test under negative marking conditions. Surveys showed that students generally preferred ET-style MCQ testing over SA-style testing. Students reported feeling more relaxed taking ET MCQ and more stressed when sitting SA tests, while disagreeing with being distracted by thinking about best tactics for scoring high. Students agreed ET testing improved their critical thinking skills. We conclude that appropriately-designed MCQ tests do not systematically discriminate between genders. We recommend careful consideration in choosing the type of MCQ test, and propose to apply negative scoring conditions to each test type to avoid the introduction of gender bias. The student experience could be improved through the incorporation of the elimination answering methods in MCQ tests via rewarding partial and full knowledge.

## Introduction

Examinations with multiple choice questions (MCQ) are increasingly used as the sole or part method of assessment of theoretical knowledge in modules with large class sizes in many bioscience degrees. MCQ tests can be used to objectively measure factual knowledge, ability and complex, high-level learning outcomes while taking advantage of a variety of different formats (reviewed in [1], [2], [3]). MCQ tests may also introduce gender bias in test performance dependent on subject test area, instruction/scoring condition and question difficulty (reviewed in [4]. A different type of MCQ consisting of questions with ‘True-False-Abstain’ answering options only has been reported to introduce significant gender bias within the medicine subject area [5].

The most commonly used MCQ test is that where the student is required to provide the Single best Answer (SA), also known as ‘Number Correct’ (NC) tests. Negative-marking in SA tests may inhibit ‘*pure guesswork’*, but students with partial knowledge are effectively forced to ‘*educated guesswork*’ whenever they are unable to decide which answer is correct. Hence, SA test score reliability is reduced. Negative marking has also received attention at University level as it may be considered unfair to risk-averse students [6]. This type of student might not feel confident enough to attempt all questions or choose an answer even if he/she were able to eliminate one or two options. In contrast to risk-averse students, students who are more prone to taking chances may still choose to gamble on the correct answer upon elimination of one or two options and subsequently these students may score a negative total mark. Student satisfaction may thus be negatively affected for both types of student behaviour in negatively-marked SA tests by their feeling of being penalised for the wrong reason and for not being able to show partial knowledge of the topic that would otherwise be rewarded in essay/short written answer-style exams [6].

Elimination testing (ET) is an alternative form of MCQ testing used to discriminate between all possible levels of knowledge [1], [7], [8], [9]. In ET, students are asked to eliminate all possible answers that they can identify as incorrect. Elimination of *y–1* incorrect options from *y* possible answers is rewarded maximally, while partial knowledge can be shown and rewarded if students are unsure about which answer is correct. Each additional incorrect answer identified by the student is rewarded with an additional positive mark while removal of the correct answer usually incurs a penalty in the ET scoring system.

The variety of responses in ET shows all possible levels of knowledge in contrast to SA testing. For ET, removing all incorrect answers indicates *full knowledge* by the student. *Partial knowledge* is shown by removal of a subset of incorrect answers. Removal of the correct answer and a subset of distractors reveal *partial misinformation*, whereas *full misinformation* is the result of eliminating the correct answer alone. Skipping over the question, i.e. no responses indicated, or removal of all options indicates *absence of knowledge*. The extent of possible responses given in the cohort may also show how difficult or easy the students perceived the question and answer options to be.

Statistically appropriate comparisons of MCQ test performance, particularly negatively-marked tests including SA and ET, and possible effects on gender bias, have not yet been performed in bioscience modules at undergraduate level according to our knowledge of the literature. This study aimed to compare student performance and possible gender bias in the life sciences between identically-worded ET and SA tests with negative marking. It also aimed to survey students on their experiences with ET and SA testing.

## Methods

### Ethics Statement

Data collected were part of routine, voluntary, formative assessments. The current manuscript describes an evaluation of those assessments as part of a teaching quality evaluation and improvement exercise, with the aim of evaluating and improving the course. Information on performance and feedback was used in anonymous form at all times. As work described here concerns an evaluation of current teaching and assessment practices, it was not necessary to obtain ethical approval. Students were made aware through providing written and oral information that the MCQ tests were voluntary and were encouraged to participate. Students were made aware that non-participation would not be logged formally in university or departmental monitoring systems nor would it lead to formal consequences.

### Data Source

Modules for which formative multiple choice questions (MCQ) tests were prepared were module PM-131 ‘Chemistry of Life’, an introductory Biochemistry module compulsory for all level 1 (L1) life sciences students enrolled on the BSc. (Hon.) degree courses Biochemistry, Medical Biochemistry, Genetics, Medical Genetics, Biology, Marine Biology, Zoology and the Joint Honours degree course Biochemistry & Genetics; and module PM-241 ‘Biochemical Techniques’, an advanced level Biochemistry module for level 2 (L2) (Medical) Biochemistry, (Medical) Genetics, and Joint Honours Biochemistry & Genetics students. Students were asked to revise materials from the syllabus taught in the first three weeks of the five-week modules. The formative MCQ test was taken in the last week of the module, allowing the students reasonably sufficient time for revision. The PM-131 and PM-241 tests were, respectively, taken on Wednesday Nov 2^nd^ and Friday Nov 4^th^ 2011, which was during regular lecture times. Student participation in the formative tests was encouraged by making available to participants free new and used textbooks, strong non-woven carrier bags in pouches, chocolates and the learning experience of sitting different types of (formative) MCQ assessments under time stress. Students received written instructions on the two types of MCQ answering methods by email well in advance of the test date. In addition, an oral instruction was given in each module during a regular lecture, and a repeat oral instruction directly before the start of each formative MCQ test.

### Data Structure and Statistical Analyses

We assessed L1 and L2 students in formative tests consisting of 25 MCQ per test, with 5 options per question, with SA- and ET-style answering sheets allowing statistically relevant comparisons. Immediately after completing the formative MCQ test, students completed survey forms, which had 42 questions (Survey S1 in File S1) with the response format in six-level Likert items: 1-Strongly agree, 2-Agree, 3-Neutral, 4-Disagree, 5-Strongly disagree, 6-Not applicable/Don’t Know. After publishing the formative MCQ test score performances and the summative January examinations results, students were surveyed again using a somewhat amended survey (‘post-survey’, Survey S2 in File S1). The descriptive statistics for each module’s participation in MCQ tests and surveys are given in Table 1.

**Table 1:** Descriptive statistics of MCQ tests and student surveys.

	L1	L2
Students drawn from degree courses	(Medical) Biochemistry, (Medical)Genetics, Biochemistry & Genetics(Joint Hon.), Biology, MarineBiology, Zoology	(Medical) Biochemistry, (Medical) Genetics,Biochemistry & Genetics (Joint Hon.)
Enrolled number of students	198	45
Number of participants (% of total)	142 (72%)	40 (88%)
Paired answer sheets	136	40
Number of Females	74	14
Number of Males	62	26
Number of MCQ per test	25	25
Number of survey respondents	142	40
Number of post-survey respondents	76	17

Both types of MCQ answer sheets were paired per participant and scanned together with the survey forms for automatic scoring using Remark Office OMR software package (v7) licensed from Gravic, which were then exported to Microsoft Office Access 2007 in anonymised (paired) form. Free text comments were collected manually. Test score performances per participant were calculated according to the scoring grid in Table 2. With this scoring grid, the total “reward” points score is equivalent to the negative score incurred if the correct answer is removed hence the average score generated by random selection of answers would be expected to be zero. Overall test scores were made available to each individual participant through the University’s Virtual Learning Environment (Blackboard).

**Table 2:** Scoring grid for SA and ET MCQ tests with negative marking.

Student indicates:	Single Answer MCQ	Elimination Answer MCQ
Correct Answer	+4 marks	−4 marks
Incorrect Answer	−1 marks	+1 mark for each answer
No Answer	0 marks	0 marks

Statistical analyses were performed using SPSS version 19. Entire (paired) datasets, rather than the means of the datasets, were used for statistical comparisons. Test performance data sets were subjected to Kolmogorov-Smirnov tests to assess whether performance scores were not significantly different from, or compatible with, a normal distribution. Subsequent tests on performance score datasets compatible with a normal distribution were a paired *t*-test to compare performance in the two types of MCQ tests per cohort, and independent *t*-tests to assess gender bias in the two types of MCQ tests per cohort and to compare performance per level of experience. An independent *t*-test was chosen for the latter as the data sets were unpaired. Two-way ANOVA tests were carried out to assess whether performance scores were influenced both by gender and type of test. Student survey responses were analysed using a non-parametric one-sample sign test to assess whether the mean of the responses were significantly different from the expected response ‘neutral’ (Likert score 3 in our survey).

## Results and Discussion

### Student Performance in SA- and ET-answering Style MCQ Tests

We assessed the MCQ test performance of level 1 (L1) and level 2 (L2) life sciences students in formative tests consisting, for each level, of 25 questions per test, with 5 answer options per question using negative marking in both types of answering to discourage guessing. Students were asked to record their answers to each question on SA and ET-style answering sheets, which were then automatically scored and analysed further. The participation rate of, respectively, 72% and 88% of students enrolled on the L1 and L2 modules allowed us to perform statistically relevant comparisons within the cohorts (Table 1). The high level of participation also suggests that students generally valued the in-kind incentives as well as the assessment experience under time stress.

A total of 142 L1 and 40 L2 students completed ET and SA MCQ answer sheets, of which 6 L1 students failed to mark one or both of the forms, resulting in, respectively, 136 and 40 paired L1 and L2 ET and SA answer sheets to be further analysed (Table 1). The overall test score performance per student per ET and SA test was calculated under negative marking scoring conditions (Table 2). L1 and L2 SA and ET overall test score performances were subjected to Kolmogorov-Smirnov tests to determine whether the test score performances were not significantly different from a normal distribution. The *P*-values for the four tests were all >0.05 (*P*-values for L1 SA and ET were 0.28 and 0.08 and for L2 SA and ET were 0.20 and 0.20 respectively), indicating that L1 and L2 SA and ET overall test score performances were compatible with a normal distribution, thus allowing further parametric testing.

**Figure 1:**
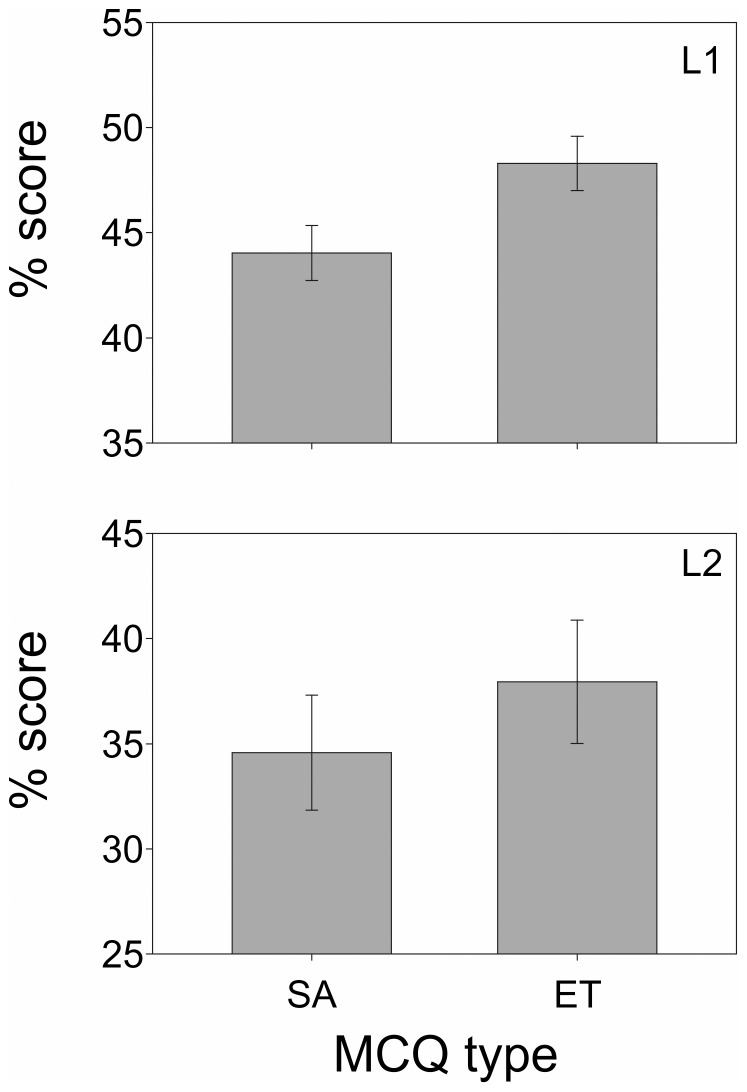
Mean (± standard error SE) of overall test score performances in L1 (top) and L2 (bottom) SA and ET style MCQ assessments.

The L1 score performance for SA and ET MCQ tests was fairly poor with averages (and standard deviation σ) of 44.0% (15.2%) and 48.3% (15.1%), respectively (Figure 1). L2 score performances were even lower with mean test scores (and standard deviation σ) of 34.6% (17.3%) and 38.0% (18.6%) for SA and ET MCQ tests, respectively, each L2 test having means constituting a Fail (Figure 1). The absolute difference in mean performance scores between L1 (MCQ novices) and L2 (experienced in SA and ET MCQs) was ∼10% for both SA and ET tests, with the novices scoring higher than L2 students with a year of experience in taking both SA and ET-style MCQ assessments. Independent *t*-tests showed that these differences were significant for both types of tests (SA: t_57.983_ = 3.123, *P* = 0.003; ET: t_55.044_ = 3.230, *P* = 0.002).

These test performance scores suggest that students may not have revised as carefully for the formative test as they would have for summative examinations. In the student surveys it was indeed admitted that participants had not revised the material adequately (Table 3). The relatively poor scores were also reflected by relatively ‘lower’ top scores in both levels for both types of test (71–80%), whereas top scores in formal MCQ examinations ranged up to 99% for both cohorts.

**Table 3:** L1 and L2 student responses immediately after sitting of the ET and SA tests on a 6 item Likert scale (survey scores).

	L1						L2					
	survey scores (n = 142)						survey scores (n = 40)					
	ET			SA			ET			SA		
Survey statements	mean	median	P	mean	median	P	mean	median	P	mean	median	P
There is no reward for random guessing	2.606	2	<0.001	2.268	2	<0.001	2.949	3	*0.721*	1.967	2	<0.001
Loosing marks for guessing detracted	3.152	3	*0.36*	3.141	3	*0.335*	2.925	3	*0.716*	2.575	2	0.018
Being able to choose more than one answerfelt very safe	2.167	2	<0.001	2.500	2	<0.001	2.025	2	<0.001	2.222	2	0.002
There is a high chance of getting answers right	2.779	2	0.023	3.555	4	<0.001	2.575	2	0.01	3.250	3	*0.093*
The answering options were confusing	3.577	4	<0.001	3.654	4	<0.001	3.525	4	0.014	3.692	4	0.002
I got distracted by thinking about the besttactics for getting a high mark	3.657	4	<0.001	3.686	4	<0.001	3.425	4	0.021	3.400	3	*0.064*
It makes you think more about your answers	2.333	2	<0.001	2.265	2	<0.001	2.462	2	0.003	2.275	2	0.003
It made me feel more relaxed, knowing thatI can get a reasonable mark	2.686	2	<0.001	3.314	4	<0.001	2.450	2	0.009	3.475	4	0.031
I could answer conservatively by hedging my bets	2.547	2	<0.001	3.788	4	<0.001	2.600	2	0.033	3.875	4	<0.001
It was a fair test	2.304	2	<0.001	2.356	2	<0.001	2.425	2	0.007	3.125	3	*0.564*
The test score will accurately reflect myknowledge	2.971	3	*0.609*	2.748	2	0.012	2.525	2	0.011	3.175	3	*0.426*
It enhanced my critical thinking skills	2.859	3	0.047	2.926	3	*0.234*	2.600	2	0.017	2.974	3	*0.846*
The questions were easy to answer	3.123	3	*0.12*	3.120	3	*0.11*	2.825	3	*0.207*	3.125	3	*0.336*
I was scared to answer some questions	3.029	3	*0.798*	2.583	2	<0.001	3.100	3	*0.558*	2.850	3	*0.404*
I was confident to answer some questions	2.139	2	<0.001	2.289	2	<0.001	2.425	2	0.004	2.425	2	0.002
It made me feel motivated	2.942	3	*0.265*	3.071	3	*0.444*	2.846	3	*0.412*	3.524	4	0.033
My stress levels were high	3.628	4	<0.001	3.223	3	0.044	3.067	3	*0.195*	3.000	3	*0.931*
It gave me confidence for the January exams	2.759	2	0.003	2.857	3	*0.087*	2.600	3	*0.063*	2.867	3	*0.362*

A *P*-score of <0.05 indicates a significant difference to the neutral response (Likert item 3); *P*-scores in italics indicate differences that are not significant to a neutral response.

The PM-131 module is the first L1 module in the first term, which is compulsory for all L1 students enrolled on any of the life science degree courses at Swansea University. The relatively poor L1 mean scores may also suggest that the novice students may not yet have adapted to University-style assessments. These students are likely to be in the transition process from A-Level style learning under guided revisions for examinations at secondary school to living away from home for the first time in the first year at university and may not yet be fully skilled and prepared for university-style ‘independent’ learning and development [10], [11]. The mean score at Fail level for both L2 tests strongly indicated that revision for the L2 formative tests was not adequate, which was admitted to in the survey (Table 3). Some students also remarked that they struggled with or misjudged the increased amount of material covered and the level of detail and knowledge required at advanced level biochemistry. Our findings are consistent with the experience of biosciences students in year 2 at Leicester University [12]. In this study, L2 students reported significant transitions with increased workload, increased learner autonomy and impacts from social and domestic issues, e.g. dealing with rental agencies and experiencing living independently off campus.

### Comparison of Performance in SA- and ET-answering Style MCQ Tests

Paired *t*-tests showed that the differences observed between SA and ET MCQ test scores were significant. The absolute difference between ET and SA test scores for L1 was 4.3% (*P*<0.001), which was ∼10% higher than the L1 SA mean score in relative terms. For L2, the absolute difference was 3.4% (*P = *0.023), which was also relatively ∼10% higher than the Sa mean score. These results show that both L1 and L2 students performed significantly better in identically worded MCQ assessments with elimination-style answering. The relatively poor average scores indicated that the majority of students may not have revised the material to the level of full knowledge for some or most of the question topics. Under these conditions, students benefit clearly by being able to show and be rewarded for partial knowledge in ET MCQ tests. ET-style MCQ tests are also of benefit to the assessor because the resulting scores are usually a more reliable indicator of a student’s knowledge [7]. This is due to students realising it is more likely to lose marks as to gain marks by random guessing in ET tests under conditions of partial knowledge on a particular topic.

Our experimental results with life sciences cohorts are in accord with the predictions from a theoretical comparison of negatively-marked SA and ET tests [7]. These comparisons predicted that liberal/free-choice MCQ tests such as ET testing reward partial knowledge more generously than conventional tests such as SA tests, in particular when a student without full knowledge can eliminate more than one correct answer, while discouraging guesswork, thereby improving the test reliability score. Our results with life sciences students in the UK are also similar to results obtained from information management students on an introductory operations management module at a Taiwanese university, which found ET scoring helpful in relation to partial knowledge and unexpected responses [13]. This may suggest that ET-style MCQ testing is also not affected by cultural differences in learning, teaching and assessing, even in different subject areas. Students on an introductory macroeconomics course also scored on average 16% higher relatively in negatively-marked ET tests over SA tests without negative marking, though the scoring calculation grid for ET was quite different from the scoring grid applied in our tests [2]. Contrasting results were obtained in a study with student applicants for Israeli universities who obtained similar scores in negatively-marked SA and ET testing averaged over four types of MCQ tests on general knowledge, general and figural reasoning and mathematical reasoning [1]. ET-style MCQ tests in general knowledge and mathematical reasoning scored higher than SA-style tests, while figural reasoning achieved the same score for both tests and general reasoning tests had a higher score for negatively-marked SA tests. Our results are similar to the Israeli study result with ET-style MCQ tests in general knowledge and mathematical reasoning.

In conclusion, it is important to consider the effect that the type of MCQ test has on assessing the real knowledge which students possess on a particular topic.

### Performance of Genders in SA and ET-answering Style MCQ Tests

**Figure 2:**
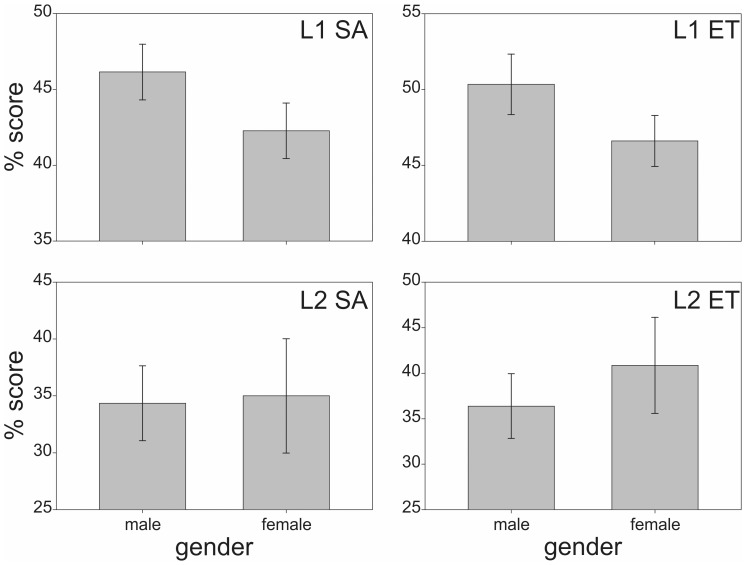
Mean (± standard error SE) of overall test score performances by gender: (top left) L1 SA MCQ, (top right) L1 ET MCQ, (bottom left) L2 SA MCQ, and (bottom right) L2 ET MCQ.

MCQ tests may introduce gender bias in test performance dependent on subject test area, instruction and/or scoring conditions and question difficulty [4]. The L1 and L2 student test score performances were grouped according to gender and averages were calculated for each MCQ test per gender per level. Independent *t*-tests were conducted to determine if gender affected overall performance in SA and ET style MCQ assessments. Figure 2 shows the results of the gender performance analyses.

The mean SA scores (and standard deviations) of L1 male and female students were 46.1% (14.4%) and 42.3% (15.8%), respectively. The absolute difference between L1 male and female SA scores was 3.8%, with L1 males scoring ∼9% higher in SA than the L1 females in relative terms. However, this difference was not statistically significant as determined by an independent *t*-test (t_134_ = 1.484, *P* = 0.140).

The L1 male and female students mean ET scores (and standard deviations) were 50.3% (15.7%) and 46.6% (14.5%), respectively. The absolute difference between L1 male and female ET scores was 3.7%, with L1 males scoring ∼8% higher in ET than the L1 females in relative terms. An independent *t*-test showed that this difference was again not statistically significant (t_134_ = 1.441, *P* = 0.152).

The mean SA scores of L2 male and female students (and standard deviations) were 34.4% (16.8%) and 35.0% (18.8%), respectively. The L2 male and female students’ mean ET scores (and standard deviations) were 36.4% (18.1%) and 40.7% (19.7%), respectively. The absolute difference between L2 male and female SA scores was 0.6%, with L2 females scoring 1.7% higher in SA than the L2 males in relative terms. The absolute difference between L2 male and female ET scores was 4.3%, with L2 females scoring ∼12% higher in SA than the L2 males in relative terms. In contrast to L1 participants, L2 female students scored on average higher than male students in both SA and ET MCQ tests, however, as for L1, the differences observed were also not statistically significant as determined by independent *t*-tests (SA: t_38_ = 0.113, *P* = 0.911; ET: t_38_ = 0.722, *P* = 0.474). Two-way ANOVA tests indicated that there was no significant interaction between the type of MCQ test and gender in either cohort (L1: *F*_1,268_ = 0.02, *P* = 0.969; L2: *F*_1,76_ = 0.202, *P* = 0.654). Our results therefore strongly suggest that both SA and ET MCQ assessments with negative marking in the life sciences subject area do not introduce statistically significant gender bias in overall test score performances at two different levels of experience.

Our results agree with more recent studies on lack of significant gender difference under highly similar MCQ testing conditions in the medical subject area [14], [15]. These and our results are in contrast to results from older studies (from the medical and other disciplines) on gender differences in MCQ performance and with results from studies using a different type of MCQ. Ricketts et al. [14] found no gender difference in SA MCQ tests with or without negative marking taken by medical students in the UK. This study also concluded that the mean score of medical students with specific learning disabilities or from ethnic minority groups was also not significantly different in their ‘properly-designed’ SA MCQ tests of medical knowledge and application. The Educational Testing Service (ETS) gender study in 1997 showed a quite small performance gap between genders in mathematics and science subjects at secondary school [16]. The gap in 1997 was much smaller than that of thirty years before the ETS study, suggesting that changes in educational experiences and campaigns effectively decreased the previously observed differences favouring male performance [16]. The ETS study did show somewhat larger gender differences in ‘self-selected’ groups taking high-stakes tests, which was reflecting primarily the wider spread of male scores, and not necessarily a wider gender gap. Our tested cohorts could be considered ‘self-selected’ through the requirements of good A-Level scores in Biology and/or Chemistry for university degree admission (specific A-Level is dependent on the entry requirements per particular degree), which suggests that the lack of gender gap found in our study has also reduced the ‘self-selected high stakes’ gender gap in the life sciences and perhaps all science, technology, engineering and mathematics (STEM) subjects. The observed absence of significant gender difference in performance in our two cohorts of life science students with different levels of experience and training reflects the continued success of changes in educational experiences and STEM campaigns in the years 1997–2011 since the findings of the ETS study. Furthermore, recent studies on the influence of gender on performance in MCQ tests in medical and surgical disciplines in Saudi Arabia reported that female students showed better overall performance in theory assessments [17]. Female performance was particularly superior in MCQ tests in the surgical disciplines, whereas the male performance was never superior in any of the MCQ tests. These findings may reflect cultural differences, leading to similar outcomes as reported in the ETS study with ‘self-selected’ high stakes tests, possibly suggesting enrolment of high-ability female Saudi-Arabian students on medical degrees.

Recent and older studies in the medical area or in other disciplines in which gender differences were found, with females scoring lower than males, often involved other types of MCQ testing and/or other scoring conditions [5], [18], [19]. A large gender difference was found in eight years of examination data of undergraduate medical students. Male medical students were 16.7 times more likely to perform better in an assessment consisting of MCQ tests with only True-False-Abstain answering options than female medical students in these cohorts [5]. In contrast, females in this study experienced advantages in course assessments, short written answer questions, and in Personal and Professional Development tasks [5]. Other studies found that the type of MCQ test and scoring conditions could render a gender bias. First year male university students from different disciplines were significantly advantaged in a SA-style MCQ test using questions from the Scholastic Aptitude Test (SAT) under scoring conditions without penalising any guessing of the correct answer, i.e. when negative marking was not applied [18]. When students were also asked to confidence-score their MCQ answers, i.e. when similar conditions to negative marking were applied, the SA test did not result in a significant gender-related difference. Our results with negatively marked SA tests are similar to the latter. A study with final year secondary school pupils showed similar results on MCQ tests on vocabulary and mental rotation topics [19]. The MCQ method favouring guessing of the answer resulted in gender difference, with males scoring higher, whereas the test taken under conditions that penalised guessing did not result in significant gender bias in test performance. The results of our negatively-marked SA tests mirror the results in the latter study.

In conclusion, when choosing a type of MCQ for formative and summative examinations it is important to consider the effect that the type of MCQ test has on performance between genders. It would be beneficial to be cautious with the adoption of MCQ tests with only True-False-Abstain answering options in assessments, unless a clear rationale can be given for this particular type of MCQ test. Care should also be applied in the choice of scoring conditions, i.e. MCQ tests with or without negative scoring, as the latter has been shown to introduce gender bias. While both ET and SA MCQ testing did not result in introduction of gender bias in our study, assessors should consider whether the test’s performance score is reflective of the student’s actual knowledge or the student’s abilities to guess in conditions of partial knowledge.

### Student Experience in SA and ET-answering Style MCQ Tests

Immediately after taking the formative MCQ tests, students were asked to complete an evaluation survey on their experiences with the SA and ET tests (Survey S1 in File S1). The survey questions allowed investigations of student attitude and emotions for each type of test, and also allowed comparisons between each test. Non-parametric one-sample sign tests were performed on the survey data to assess whether the student responses were significantly different from the ‘neutral’ score (Likert score 3). The Mean and Median scores for the L1 and L2 student responses are shown in Table 3, which also includes the *P*-value to assess significant differences from the expected ‘neutral’ score.

With regards to technical aspects of the tests, L1 and L2 students agreed that there was no reward for random guessing in both tests, suggesting full awareness of the scoring conditions under negative marking. L1 students were on average neutral in their responses to whether loosing marks distracted or not from answering the questions in ET style, while L2 students agreed it was distractive under SA, but not under ET testing conditions. Most students disagreed with the statement that they got distracted by thinking about best tactics for getting a high mark in both types of tests. Students also disagreed with the statement that answering options were confusing, suggesting they had understood the test questions and answering options. In the free-text remarks one student admitted to not understanding that the same questions needed to be answered in two formats, while one student admitted that ‘*false’* questions (e.g. Which one of the following five statements on X is *false*?) were confusing. Students agreed with the statement that they had answered conservatively in ET tests by hedging their bets, whereas they disagreed with this in SA tests. Most students agreed that both negatively marked SA and ET tests were fair, which is in contradiction to responses from economics students on negative marking in SA-style MCQ examinations [20]. Both cohorts felt there was a high chance for getting answers right in ET testing, whereas they disagreed (L1) or were neutral (L2) with a high chance of getting answers right in SA testing. L1 and L2 students were neutral in their response to whether the questions were easy to answer for both tests.

When asked about emotional experiences with the MCQ tests, students agreed that ET testing made them feel more relaxed by knowing they could achieve a reasonable mark, while they disagreed with this for SA testing. This feeling was enhanced by the students’ general agreement that selecting more answers felt safe, while choosing one answer felt risky. Students felt neutral towards being scared to answer questions ET-style, whereas L1 students agreed to feeling scared to answer in SA format. Yet, students agreed they were confident in answering questions in both ET and SA tests. L1 students disagreed with their stress levels being high in ET testing, while disagreeing (with a very slight significant difference) on high stress levels for SA testing. L2 students were neutral for high stress levels for both tests. When asked to directly compare stress levels for both tests, both cohorts agreed they were more stressed with SA testing than with ET testing. The L2 students were neutral for both tests whether it gave them confidence for the upcoming summative, formal examinations, whereas L1 students agreed that the ET test had given them confidence for the upcoming examinations, whereas they responded neutral in relation to SA testing.

With regards to study skills and knowledge levels, students in general agreed that both types of tests made them think more about the answers. L1 and L2 both agreed ET testing enhanced their critical thinking skills, whereas they were neutral to enhanced critical thinking for SA testing. L1 and L2 students thought that the SA and ET score, respectively, would accurately reflect their knowledge, whereas they were neutral to this question for, respectively, ET and SA testing, which could be explained by a difference in experience of taking ET and SA-style tests. Students responded mostly neutral to the question of feeling motivated by either type of test.

Surveying for direct comparisons directly after sitting the tests (Table 4), both cohorts thought ET testing would lead to higher scores and similarly disagreed with the statement that SA testing would lead to higher scores than ET testing. They were however more tentative, i.e. neutral, in their responses to whether SA testing would lead to lower scores than ET testing. After taking everything into consideration immediately after sitting the tests, both cohorts strongly preferred ET testing and did not prefer SA testing.

**Table 4:** L1 and L2 student responses on comparative statements on ET and SA MCQ testing on a 6 item Likert scale immediately after sitting the test (survey scores) and after receiving test and formal examination results (post survey scores).

	L1						L2					
	survey scores (n = 142)			post survey scores (n = 76)			survey scores (n = 40)			post survey scores (n = 17)		
Comparison of ET and SA answering options	mean	median	P	mean	median	P	mean	median	P	mean	median	P
SA testing will lead to a higher score compared to ET	3.661	4	<0.001				3.700	4	0.001			
SA testing will lead to a lower score compared to ET	2.896	3	*0.176*				2.567	3	*0.06*			
ET will lead to a higher score compared to SA	2.518	2	<0.001				2.667	2.5	*0.15*			
There is a higher chance of getting answers right with ET than with SA	2.526	2	<0.001				2.467	2	0.017			
I was more stressed with SA testing than with ET	2.661	2	0.008				2.167	2	0.001			
After taking all aspects into consideration, I prefer SA testing	3.530	4	<0.001	2.577	2	0.015	3.600	4	0.03	4.056	4	0.002
After taking all aspects into consideration, I prefer ET testing	2.600	2	0.002	3.282	3	*0.075*	2.333	3	*0.06*	1.667	1.5	<0.001
The results from both MCQ tests were as I expected				2.870	3	*0.238*				2.333	2	0.018
I expected a higher mark for the elimination test				3.680	4	<0.001				2.167	2	0.002
I expected a higher mark for the single answer test				3.539	3.5	<0.001				3.278	3	*0.284*
I expected to do equally as well for both MCQ tests				2.889	4	0.003				3.539	3	*0.714*
I prefer to be rewarded for knowing or guessing the answers exactly				2.474	2	<0.001				3.611	4	0.047
I prefer to be rewarded for demonstrating my partial and full knowledge				2.526	2	<0.001				1.667	2	<0.001
My revision for the voluntary mcq tests was adequate				3.641	4	<0.001				3.500	4	*0.146*
I should have revised more for the voluntary mcq test				2.308	2	<0.001				2.556	2.5	*0.174*

A *P*-score of <0.05 indicates a significant difference to the neutral response (Likert item 3); *P*-scores in italics indicate differences that are not significant to a neutral response.

### Changes in Student Experience

The students were also surveyed after they had received their scores for the formative tests and after receiving scores for formal summative examinations, which included MCQ tests in both formats. This ‘Post-Survey’ (Survey S2 in File S1) enables comparisons of views on both types of tests after the students had gained more experience with it and had more time to reflect on their performance and experiences in either test. The number of post-survey respondents was (more than) halved, when compared to the numbers of participants in the MCQ tests (Table 1), indicating that the incentives helped to raise the participation rate in the original test.

The respondents in the Post-survey changed their opinions somewhat on some of the statements (Tables 4 and 5). The most notable changes in opinion are discussed. Most notably, L1 students changed their opinion most and now agreed with the statement that they got distracted by thinking about best tactics for getting a high mark in both types of tests, whereas L2 students disagreed with this statement. L2 students changed to a neutral response to answering conservatively through bet hedging for both tests, while L1 changed from disagreed to agreed with answering conservatively through bet-hedging for SA test (while remaining agreed for ET). Both cohorts changed their response from agreed to neutral on high chances of getting answers right in ET testing, suggesting students felt that ET testing was more difficult than perceived originally. L1 students now agreed with high stress levels during SA testing, while responding neutral to experiencing high stress levels in ET testing. The L2 student response showed a slightly significant change from neutral to disagreeing with high stress levels for ET testing. L1 students went from agreed to neutral on ET testing making them think more about their answers, whereas for SA testing L1 students now disagreed that it made them think more. Both L1 and L2 responded from agreed to neutral on enhanced critical thinking by ET testing.

**Table 5:** L1 and L2 student responses to ET and SA MCQ testing after having received test and formal examination results (post survey scores) on a 6 item Likert scale.

	L1						L2					
	post survey scores (n = 76)						post survey scores (n = 17)					
	ET			SA			ET			SA		
Survey statements	mean	median	P	mean	median	P	mean	median	P	mean	median	P
There is no reward for random guessing	2.077	2	<0.001	2.321	2	<0.001	2.667	2.5	*0.175*	2.222	2	0.002
Loosing marks for guessing detracted	2.641	2	0.002	2.949	3	*0.465*	2.944	3	*0.942*	3.167	3	*0.499*
There is a high chance of getting answers right	3.308	3	*0.054*	3.766	4	<0.001	2.833	3	*0.49*	3.471	3.5	0.033
The answering options were confusing	2.987	3	*0.843*	3.474	3.5	<0.001	4.167	4.5	0.002	3.778	4	0.023
I got distracted by thinking about the best tactics for getting a high mark	2.397	2	<0.001	2.680	2	0.016	3.765	4	0.022	3.833	4	0.007
It makes you think more about your answers	2.810	3	*0.201*	3.436	4	0.001	2.389	2	0.047	2.889	3	*0.617*
It made me feel more relaxed, knowing that I can get a reasonable mark	2.641	2.5	0.001	3.846	4	<0.001	2.278	2	0.028	3.444	3	*0.12*
I could answer conservatively by hedging my bets	2.346	2	<0.001	2.423	2	<0.001	2.944	3	*0.782*	3.389	3	*0.118*
It was a fair test	2.974	3	*0.606*	3.416	3	0.025	2.056	2	0.001	2.944	3	*0.82*
The test score will accurately reflect my knowledge	2.923	3	*0.276*	3.346	3	0.021	2.278	2	0.016	2.444	2	0.019
It enhanced my critical thinking skills	3.051	3	*0.589*	3.321	3.5	0.013	2.722	3	*0.284*	3.111	3	*0.564*
The questions were easy to answer	3.397	3	0.006	3.115	3	*0.507*	2.778	3	*0.285*	3.167	3	*0.439*
I was scared to answer some questions	2.731	2	0.038	3.321	4	0.016	3.556	3	*0.075*	2.722	3	*0.197*
I was confident to answer some questions	3.397	3.5	0.003	2.974	3	*0.815*	1.889	2	<0.001	2.833	3	*0.444*
My stress levels were high	3.077	3	*0.64*	2.577	2	0.004	3.611	4	*0.057*	2.944	3	*0.854*
It gave me confidence for the January exams	3.103	3	*0.63*	3.359	3.5	0.009	2.722	3	*0.265*	2.889	3	*0.589*
It was good preparation for the real ET exams in January	2.872	3	*0.316*	3.039	3	*0.822*	2.222	2	0.023	2.722	3	*0.349*
Knowing my score now, I should have eliminated less answers as I was guessing too much	3.885	4	<0.001	3.103	3	*0.619*	3.278	3	*0.222*	3.278	3	*0.372*

A *P*-score of <0.05 indicates a significant difference to the neutral response (Likert item 3); *P*-scores in italics indicate differences that are not significant to a neutral response.

On reflecting upon the scores achieved in the formative tests, most students responded neutrally to the statement that they should have eliminated fewer answers or left more questions unanswered for, respectively, ET and SA testing. Most students disagreed that their revision had been adequate for the formative tests, and agreed indeed that they should have revised more.

Upon having had the time to reflect on direct comparisons between the tests, the L1 student response differed from the L2 response (Table 4). The L2 cohort agreed their scores were as expected, whereas L1 students responded neutral. The L1 students had expected to do equally well in either test and disagreed that either test would score higher than the other test. In contrast, L2 students agreed that they had expected a higher score for ET tests than for SA tests, but responded neutral to doing equally well in either test.

L1 students preferred to be rewarded for both knowing or guessing the answer exactly AND for demonstration of partial and full knowledge rather than guessing, which may suggest two camps of thoughts exist within the cohort, possibly reflecting the wider range of degrees participating students were enrolled on. L2 students were very explicit in showing high preference for being rewarded for demonstration of partial and full knowledge rather than guessing, and disliked being rewarded for knowing or guessing the answer exactly. After taking everything into consideration after sitting the tests and experiencing further summative examinations, L2 students did not prefer SA testing and (strongly) preferred ET testing. L1 students preferred SA testing and were neutral to ET testing. This slight difference possibly reflects the difference that exists in experience between cohorts with taking ET-style MCQ tests, with L2 students taking most of their summative examinations with MCQ tests in ET format and thus having more experience with ET testing than SA testing.

### Conclusions

We conclude that appropriately-designed multiple-choice tests of biochemical knowledge do not systematically discriminate between genders. We recommend careful consideration in choosing the type of MCQ test in relation to performance and gender bias, and propose to apply negative scoring conditions to each test type. The student (learning) experience could be improved through the incorporation of the elimination answering methods in MCQ tests via rewarding partial and full knowledge.

## Supporting Information

File S1 **Questions used in the student feedback Survey (S1) and Post-Survey (S2).**
(DOCX)
