# Supplementary material for: Negatively-Marked MCQ Assessments That Reward Partial Knowledge Do Not Introduce Gender Bias Yet Increase Student Performance and Satisfaction and Reduce Anxiety
Source: PLoS One. 2013 Feb 20;8(2):e55956. doi: 10.1371/journal.pone.0055956 (PMC3577794; doi:10.1371/journal.pone.0055956)
Supplement: File S1 — Questions used in the student feedback Survey (S1) and Post-Survey (S2). (DOCX) [file pone.0055956.s001.docx]

SURVEY S1 QUESTIONS

* questions edited/removed in the Post-Survey S2 due to space constraints on the survey form

**Elimination Answering**

- * Being able to choose more than one answer felt very safe
- There is a high chance of getting answers right
- The answering options were confusing
- I got distracted by thinking about the best tactics for getting a high mark
- It makes you think more about your answers
- It made me feel more relaxed, knowing that I can get a reasonable mark
- I could answer conservatively by hedging my bets
- It is a fair test
- Loosing marks for guessing detracted from the legitimate marks for knowing the right answers to some questions
- The test score will accurately reflect my knowledge
- It enhanced my critical thinking skills
- The questions were easy to answer
- I was scared to answer some questions
- I was confident to answer some questions
- * It made me feel motivated
- My stress levels were high
- There is no reward for random guessing
- It gave me confidence for the January exams

**Single Answer**

- * Having to choose just one answer felt very risky
- There is a high chance of getting answers right
- The answering options were confusing
- I got distracted by thinking about the best tactics for getting a high mark
- It makes you think more about your answers
- It made me feel more relaxed, knowing that I can get a reasonable mark
- I could answer conservatively by hedging my bets
- It is a fair test
- Loosing marks for guessing detracted from the legitimate marks for knowing the right answers to some questions
- The test score will accurately reflect my knowledge
- It enhanced my critical thinking skills
- The questions were easy to answer
- I was scared to answer some questions
- I was confident to answer some questions
- It made me feel motivated
- My stress levels were high
- There is no reward for random guessing
- It gave me confidence for the January exams

**Comparison of the two answering options**

- * Single answering testing will lead to a higher score compared to elimination
- * Single answer testing will lead to a lower score compared to elimination
- * Elimination testing will lead to a higher score compared to single answer testing
- * There is a higher chance of getting answers right with the elimination testing than with single answer testing
- * I was more stressed with single answer testing than with elimination testing
- After taking all aspects into consideration, I prefer single answering testing
- After taking all aspects into consideration, I prefer elimination testing

POST-SURVEY S2 QUESTIONS

*: questions added or edited

**Elimination Answering**

- The answering options were confusing
- There was a high chance of getting answers right
- I got distracted by thinking about the best tactics for getting a high mark
- It made you think more about your answers
- It made me feel more relaxed, knowing that I could get a reasonable mark
- I could answer conservatively by hedging my bets
- It was a fair test
- Loosing marks for guessing detracted from the legitimate marks for knowing the right answers to some questions
- The test score accurately reflected my knowledge at the time
- It enhanced my critical thinking skills
- The questions were easy to answer
- I was scared to answer a question
- I was confident to answer a question
- My stress levels were high
- There was no reward for random guessing
- * It was good preparation for the real ET exams in January
- It made me feel confident for the January exams
- * Knowing my score now, I should have eliminated less answers as I was guessing too much

**Single Answer**

- The answering options were confusing
- There was a high chance of getting answers right
- I got distracted by thinking about the best tactics for getting a high mark
- It made you think more about your answers
- It made me feel more relaxed, knowing that I could get a reasonable mark
- I could answer conservatively by hedging my bets
- It was a fair test
- Loosing marks for guessing detracted from the legitimate marks for knowing the right answers to some questions
- The test score accurately reflected my knowledge at the time
- It enhanced my critical thinking skills
- The questions were easy to answer
- I was scared to answer a question
- I was confident to answer a question
- My stress levels were high
- There was no reward for random guessing
- * It was good preparation for the real SA exams in January
- It made me feel confident for the January exams
- * Knowing my score now, I should have left more questions unanswered as I was guessing too much

**Comparison of answering options/other**

- * The results from both MCQ tests were as I expected
- * I expected a higher mark for the elimination test
- * I expected a higher mark for the single answer test
- * I expected to do equally as well for both MCQ tests
- I prefer single answer testing
- I prefer elimination testing
- * I prefer to be rewarded for knowing or guessing the answers exactly even though there is a penalty for answering or guessing incorrectly
- * I prefer to be rewarded for demonstrating my partial and full knowledge then guessing what the right answer is
- * My revision for the formative mcq tests was adequate
- * I should have revised more for the formative mcq test
